# Challenges and opportunities to delivering cardiac imaging training: a national survey by the Italian college of cardiac radiology

**DOI:** 10.1186/s13244-021-01076-5

**Published:** 2021-09-27

**Authors:** Marco Gatti, Carlo Liguori, Giuseppe Muscogiuri, Riccardo Faletti, Serena Dell’Aversana, Patrizia Toia, Gianluca De Rubeis, Paolo Di Renzi, Vincenzo Russo, Gesualdo Polizzi, Nicola Galea, Antonio Esposito, Marco Francone

**Affiliations:** 1 Radiology Unit, Department of Surgical Sciences, University of Turin, Turin, Italy; 2 Radiology Unit, ASL Napoli1Centro-Ospedale del Mare, Naples, Italy; 3 Department of Radiology, IRCCS Istituto Auxologico Italiano, San Luca Hospital, University Milano Bicocca, Milan, Italy; 4 Department of Radiology, Ospedale S. Maria Delle Grazie - ASL Napoli 2 Nord, Pozzuoli, Italy; 5 Department of Biomedicine, Neurosciences and Advanced Diagnostics - BIND, University of Palermo, Palermo, Italy; 6 Department of Radiological, Oncological and Pathological Sciences, Sapienza University of Rome, Rome, Italy; 7 Department of Diagnostic Imaging, AO San Camillo/Forlanini, Rome, Italy; 8 Radiology Unit, “San Giovanni Calibita” Fatebenefratelli Hospital, Isola Tiberina, Rome, Italy; 9 U.O, Radiologia Cardio-Toracica, Polo Cardio-Toraco-Vascolare, Policlinico S.Orsola-Malpighi, Bologna, Italy; 10 Unit of Radiodiagnostics II, University Hospital Policlinico “G. Rodolico - San Marco”, Catania, Italy; 11 Department of Experimental Medicine, “Sapienza” University of Rome, Rome, Italy; 12 Experimental Imaging Center, IRCCS San Raffaele Scientific Institute, Milan, Italy; 13 School of Medicine, Vita-Salute San Raffaele University, Milan, Italy; 14 Department of Biomedical Sciences, Humanitas University, Via Rita Levi Montalcini 4, 20072 Pieve Emanuele, Milan, Italy; 15 IRCCS Humanitas Research Hospital, via Manzoni 56, 20089 Rozzano, Milan, Italy

**Keywords:** Cardiac imaging, Coronary computed tomography angiography (CCTA), Cardiac magnetic resonance (CMR), Radiology residency, Education

## Abstract

**Background:**

Delivering consistent levels of training in cardiac imaging to radiologist is of pivotal importance because of the increasing clinical indications to coronary computed tomography angiography (CCTA) and cardiac magnetic resonance (CMR). Our study sought to capture the heterogeneity of cardiac imaging training programs and to explore residents’ vision on cardiac imaging both in the present and in the future.

**Methods:**

Two web-based surveys were created. The first was administered to all chief residents from the 42 University Hospitals within Italy, aiming to explore the local educational offer in cardiac imaging. The second was administered via social media to all Italian residents, including questions about their overall vision regarding cardiac imaging.

**Results:**

42/42 University Hospitals responded to the first survey and 235 residents to the second. There was at least a 64-slice CT scanner and a 1.5 T MR scanner per center. In the majority of sites, the weekly routine consisted of more than 10 CCTA and more than 5 CMR. Approximately, half of the centers used advanced CCTA and CMR techniques. The majority of the interviewed resident (94%) perceived cardiac imaging training to be moderately to very important, while requirement for external educational resources was advocated in 25% of the cases.

**Conclusion:**

Our survey highlighted a significant awareness of radiology residents regarding the importance of cardiac imaging in their training curriculum. All centers met the technical requirements for cardiac imaging, limiting its use to basic applications in around half of cases. Implementation of an educational network might be the key for supporting the growth of this subspecialty field.

**Supplementary Information:**

The online version contains supplementary material available at 10.1186/s13244-021-01076-5.

## Keypoints

- Minimum standard requirements to perform CCTA and CMR are fully made available in all the Italian University hospitals.
- Cardiac imaging education is limited to basic applications in about the half of the cases.
- There are significant differences in training opportunities between CCTA and CMR.
- Cardiac radiology is considered highly relevant by most of the residents, in view of their future career paths.
- The training program on cardiac imaging should be improved in a quarter of the Italian post-graduate school of radiology, according to the answers of the attending residents.

## Background

Cardiac imaging accounts for approximately 30% of all Medicare-related imaging procedures, and it is growing faster than any other field of radiology [1, 2].

It has been recognized as a radiological subspecialty since the early 2000s and, subsequently, became an integral part of every radiologist’s curriculum. European Society of Radiology (ESR) ‘s training framework [3], for instance, is structured into a progressive spiral learning model which starts from medical school (Undergraduate Radiological Education), progresses during residency (level I–II) and concludes with a dedicated subspecialty training to be held after radiological board-certification (level III) [4]. Final step is the recognition of a certificate to be provided by the European Board of Cardiovascular Radiology (EBCR) [5], which formally certifies the ability of a radiologist to perform, interprets and reports coronary computed tomography angiography (CCTA) and cardiac magnetic resonance (CMR) independently.

Italian radiology residency program lasts 4 years. Six months are fully dedicated to cardiac and interventional training. This model is progressively adapting to European standards provided by the European Union of Medical Specialists (Union Européenne des Médecins Spécialistes—UEMS) [6].

Delivering consistent levels of education in cardiac imaging is essential to provide adequate health care services and to improve cardiac health, due to the recognized value of cardiac imaging for early diagnosis, disease phenotyping and prognostic stratification, while supporting clinical decision-making process [7–10].

Mastering advanced cardiac imaging competencies require a combination of computed tomography (CT) and magnetic resonance (MR) physics knowledge, together with a deep understanding of cardiac anatomy, physiology and pathophysiology.

As such, the acquisition of advanced skill sets is considered a longitudinal, if not a life-long, learning process, which is also strongly dependent on the availability of up-to-date scanners’ technology for clinical practice.

Our study has sought to capture the heterogeneity of cardiac imaging training programs by means of a national survey submitted to all Italian academic medical Institutions, so as to understand the residents’ vision regarding both the present and the future of cardiovascular imaging and the possible training offers that each school provides. This survey was, in particular, aimed at figuring out both the strength and weaknesses of radiology residency programs, while identifying the corner stone for the next future of cardiac imaging in Italy.

Promoter of the initiative was the young club of the Executive Committee of the Italian Cardiac Radiology Society (SIRM-Cardioradiologia, also defined “Cantera” Group). The “Cantera” project was built according to instructions from the European Society of Cardiovascular Radiology Young Club (ESCR YC) [11]. The mission of the “Cantera” is to involve and promote the cardiovascular imaging among young radiologists and residents.

## Methods

### First part

The first part of the survey was sent by e-mail to all chief residents from the 42 University Hospitals of Italy. It contained 31 multiple-choice (with 3–5 options each) questions on the educational offerings of the radiology University departments in Italy. Twenty-three out of thirty-one (74%) questions were about presence and management of CCTA and CMR diagnostic services in their centers. The questions concerned the scanner and the scanning protocol, the number of examinations performed, the number and the experience of radiologists, as well as the number of patients. Eight out of thirty-one (26%) questions focused on the organization of cardiac imaging training, which is related to the presence/absence and the number of dedicated cardiac imaging lectures, the possibility to attend conferences, the presence and obligation of cardiac imaging rotation during residency, the involvement of the trainees during reporting, as well as the presence of a dedicated cardiac imaging case logbook.

### Second part

The second part of the survey was administered via social media (Facebook, Menlo Park, California, USA) to all residents through the web link (https://www.facebook.com/cardio.radiologia/). The total number of followers of the official social web platforms (Facebook, Instagram, LinkedIn) of the Italian Cardiac Radiology Society amounted to 1642 (of which about 500 are radiology residents) at the time of writing the manuscript. All the people included in the survey confirmed the “status” of resident with a certification of their own university. This part included 10 questions concerning the importance/quality of their education in cardiac imaging and the possibility and usefulness of doing research in this field, as well as the relation between radiologists and cardiologists in their universities, their feeling about the future of cardiac imaging and their personal interest in performing cardiac imaging in the upcoming years. Radiology residents’ opinions were evaluated using a 5-point Likert’s scale.

The web-based survey was created using Google Forms (Google, Menlo Park, California, USA). The answers to the questionnaires were automatically downloaded (.csv file) and entered in Microsoft Excel (Microsoft, Redmond, Washington, U.S.) spreadsheets for tabulation. A part of the survey was based on previously published data [12, 13]. The full text of the survey is reported in Additional file 1*.*

## Results

### Survey part 1: CCTA and CMR diagnostic services, training programs and opportunities

**Fig. 1:**
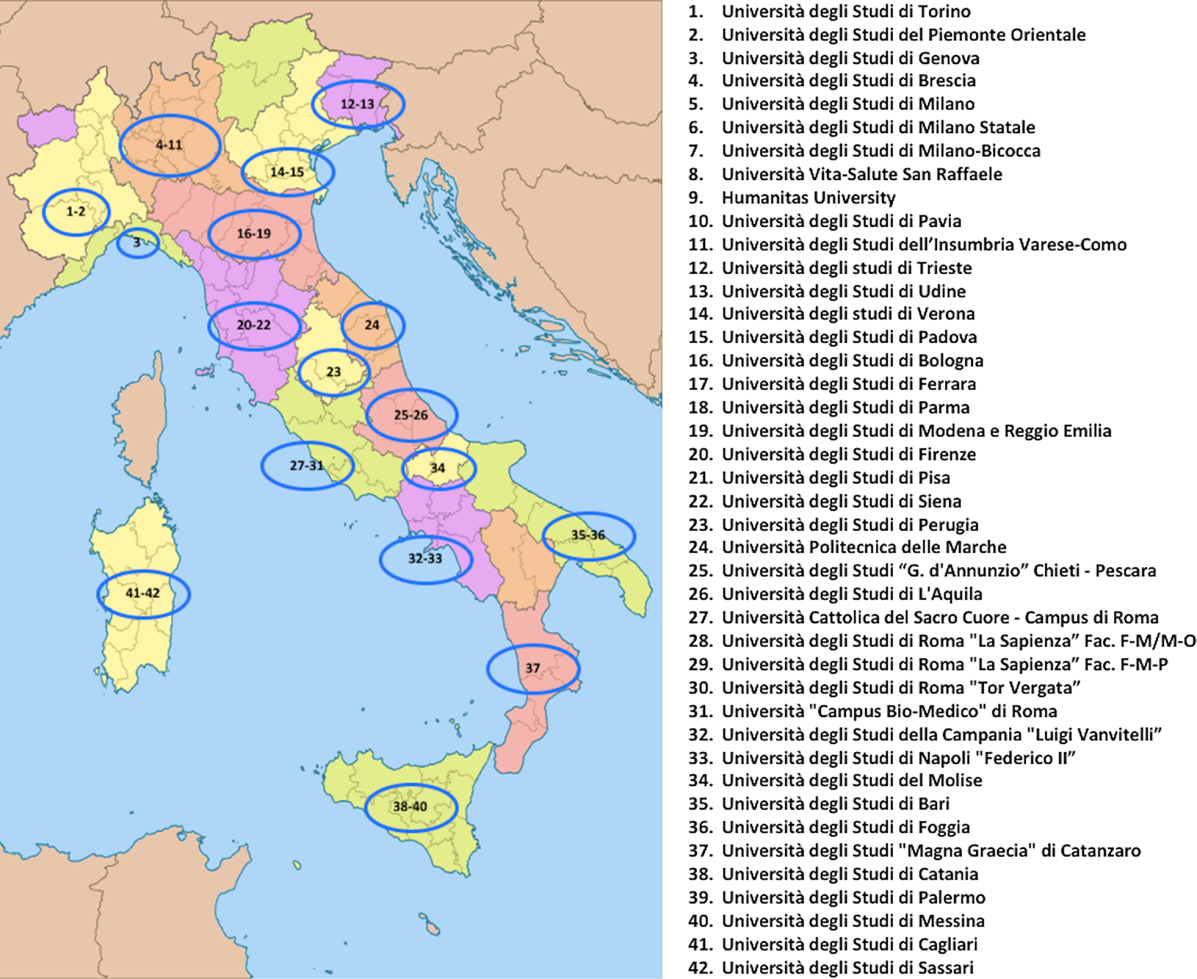
Schematic distribution of participating sites in the Italian national territory

First part of the survey received responses from 100% (42/42) of the University centers involved. The schematic distribution of participating sites in the Italian national territory is shown in Fig. 1.

### CCTA results

In every center, there was at least a 64-slice scanner CT. In 38% (16/42) of the centers, there was a whole-heart coverage CT scanner. The amount of CCTA scans performed in a single center ranges from 1 to 10 per week in 50% (21/42) of the centers, from 11 to 20 or more than 20 in 24% of them (12/42). 40% (17/42) of the centers used advanced CCTA techniques (i.e., dual-energy CT, CT Perfusion, Fractional Flow Reserve CT and late iodine enhancement).

The radiologist reported CCTA in 90% (38/42) of the cases, while in 10% of centers (4/42), a combined radiology-cardiology consensus was also reported. Radiologists who were able to report CCTA were ≤ 24% of the staff members in most of the centers (83%). More than three radiologists are involved in CCTA workflow in 31% of the centers interviewed. Furthermore, the majority of radiologists administer drugs during CCTA.

Table 1 details the answers to the 11 questions regarding CCTA.

**Table 1:** Questions and answers on CCTA diagnostic services

Coronary computed tomography angiography					
Survey item	Answer 1: n° (%)	Answer 2: n° (%)	Answer 3: n° (%)	Answer 4: n° (%)	Answer 5: n° (%)
CCTA scanner (n° slice)	64-slice: 6 (14%)	128-slice: 19 (45%)	256/320-slice: 16 (38%)	No answer: 1 (3%)	
N° of CCTA scan per week	0: 1 (2%)	1–10: 21 (50%)	11–20: 10 (24%)	> 20: 10 (24%)	
N° of pre-TAVI CT scan per week	0: 3 (7%)	1–5: 19 (45%)	6–10: 12 (29%)	> 10: 8 (19%)	
Use of advances cardiac CCTA techniques	None: 25 (60%)	Dual-energy: 9 (21%)	Delayed enhancement: 6 (14%)	Stress perfusion: 5 (12%)	FFR-CT: 3 (7%)
Patients scanned	Outpatients: 2 (5%)	Inpatients: 3 (7%)	Outpatients + Inpatients: 25 (59%)	Outpatients + Inpatients + Emergency Room: 12 (29%)	
N° of radiologist doing CCTA	0: 1 (2%)	1: 3 (7%)	2: 12 (29%)	3: 13 (31%)	> 3: 13 (31%)
% of radiologist doing CCTA	0–24%: 35 (83%)	25–49%: 4. (10%)	50–74%: 0 (0%)	75–100%: 3 (7%)	
Senior radiologist experience	< 5 years: 6 (14%)	> 5 years: 36 (86%)			
Administration of medication during CCTA	Radiologist: 30 (71%)	Radiologist + Cardiologist: 8 (19%)	No administration: 4 (10%)		
Who report CCTA?	Radiologist: 38 (90%)	Radiologist + Cardiologist: 4 (10%)	Cardiologist: 0 (0%)		
Are there radiology technicians dedicated to CCTA?	Yes: 25 (60%)	No: 17 (40%)			

CCTA: coronary computed tomography angiography; TAVI: transcatheter aortic valve implantation; FFR-CT: fractional flow Reserve-computed tomography

### CMR results

CMR was performed with a 1.5 T MR scanner in the majority of centers (81%, 34/42). In more than 80% of the cases (35/42), scanners were equipped with at least an 8-channel radiofrequency surface coil. The number of CMR examinations per week ranged from 1 to 10 in about three-quarters of centers. 55% (23/42) of the centers used advanced CMR imaging techniques (i.e., mapping sequences, stress CMR and 4D-flow), with the mapping sequences being the most employed (43%, 9/23).

In the vast majority of cases: (91%, 38/42) radiologists who reported CMR are only a small fraction of the entire radiological working group, representing ≤ 24% of the entire staff. In 19% (8/42) of the sites, there was a radiology-cardiology combined reading of the cases; in none of the centers, cardiologist reported CMR examinations independently. At least two radiologists are involved in CMR workflow in 86% (36/42) of the centers interviewed.

Table 2 details the answers to the 12 questions regarding CMR.

**Table 2:** Questions and answers on CMR diagnostic services

Cardiovascular magnetic resonance					
Survey item	Answer: n° (%)	Answer: n° (%)	Answer: n° (%)	Answer: n° (%)	Answer: n° (%)
MR scanner (field)	< 1.5 T: 0 (0%)	1.5 T: 34 (81%)	3 T: 7 (17%)	No answer: 1 (2%)	
Type of coil (n° of coil elements)	< 8: 3 (7%)	8: 7 (17%)	16: 12 (29%)	32: 16 (38%)	No answer: 4 (9%)
n° of CMR per week	0: 0 (0%)	1–5: 16 (38%)	6–10: 15 (36%)	> 10: 9 (21%)	No answer: 2 (5%)
n° of MR-angiography per week	0: 6 (14%)	1–5: 25 (60%)	6–10:6 (14%)	> 10: 5 (12%)	
Patients scanned	Outpatients only: 0 (0%)	Inpatients only: 2 (5%)	Outpatients + Inpatients: 40 (95%)		
Use of advances CMR techniques	None: 19 (45%)	Mapping Sequences: 18 (43%)	Stress Perfusion: 11 (26%)	4D-Flow: 5 (12%)	
n° of radiologist doing CMR	0: 1 (2%)	1: 5 (12%)	2: 18 (43%)	3: 10 (24%)	> 3: 8 (19%)
% of radiologist doing CMR	0–24%: 38 (91%)	25–49%: 1 (2%)	50–74%: 1 (2%)	75–100%: 2 (5%)	
Senior radiologist experience	< 5 years: 6 (14%)	> 5 years: 36 (86%)			
Collaboration with cardiologist during acquisition of CMR	Yes: 10 (24%)	No: 31 (74%)	No answer: 1 (2%)		
Who report CMR?	Radiologist: 34 (81%)	Radiologist + Cardiologist: 8 (19%)	Cardiologist: 0 (0%)		
Are there radiology technicians dedicated to CMR?	Yes: 26 (62%)	No: 16 (38%)			

CMR: cardiac magnetic resonance

### Radiology resident program

Dedicated cardiac imaging programs for residents were reported in the radiology course curriculum for most of the sites (60%, 25/42). 41% (17/42) of the centers allowed the residents to attend more than 15 h of radiological congress on cardiac imaging per year. A proper cardiac imaging training, intended as a mandatory rotation period of at least 6 months during residency, was provided in only 26% of interviewed centers. This percentage drops further to 10% if teaching program is meant to be considered by a combination of case-reading and formal front teaching lectures for > 10 h/year.

Table 3 details the answers to the eight questions regarding the training of radiology residents.

**Table 3:** Questions and answers on the training of radiology residents

Training of radiology resident					
Survey item	Answer: n° (%)	Answer: n° (%)	Answer: n° (%)	Answer: n° (%)	Answer: n° (%)
Availability of didactic lectures	No: 14 (33%)	CCTA: 2 (5%)	CMR: 0 (0%)	CCTA + CMR: 25 (60%)	No answer: 1 (2%)
Didactic lectures (hours per year)	≤ 5: 29 (69%)	6–10: 7 (17%)	11–15: 4 (10%)	> 15: 0 (0%)	No answer: 2 (4%)
Case conference (hours per year)	≤ 5: 14 (33%)	6–10: 6 (14%)	11–15: 3 (7%)	> 15: 17 (41%)	No answer: 2 (5%)
Cardiac rotation	Mandatory: 11 (26%)	Optional: 24 (57%)	Does not exist: 5 (12%)	Does not exist, but possibility to do in another center: 1 (2%)	No answer: 1 (3%)
Cardiac rotation (modalities)	CCTA + CMR: 29 (69%)	CCTA: 2 (5%)	CMR. 1 (2%)	Not exist: 9 (21%)	No answer: 1 (3%)
% of resident dedicated to cardiac imaging	0–24%: 34 (81%)	25–49%: 4 (10%)	50–74%: 0 (0%)	75–100%: 4 (9%)	
Teaching method (regarding the reporting)	Alone with correction: 26 (62%)	Assisted reading: 10 (24%)	Just watching report: 4 (10%)	No answer: 2 (4%)	
Presence of cardiac imaging cases logbook	Yes: 21 (50%)	No: 21 (50%)			

CCTA: coronary computed tomography angiography; CMR: cardiac magnetic resonance

### Survey Part 2: Radiology residents’ opinions

We received responses from 235 residents out of the approximately 500 (47%) of them following the official social web platforms: 67 (29%) in the first year of residency, 64 (27%) in the second, 54 (21%) in the third and 50 (23%) in their last.

94% of residents (220/235) reported that cardiac imaging training is at least moderately relevant in their training program. However, about a quarter of them realized the lack of an adequate training in CCTA (28%, 66/235) and in CMR (30%, 70/235). Regarding their careers, 74% (174/235) of residents opine they may do cardiac imaging in their future, and more than a half (63%, 147/235) believe that most of the cardiac imaging will have a combined radiology-cardiology reading.

Table 4 details the answers to the 10 questions regarding the radiology residents’ opinion.

**Table 4:** Survey Sect. 2: questions and answers about the radiology resident’s opinion

Radiology resident opinion					
Survey item	Answer: n° (%)	Answer: n° (%)	Answer: n° (%)	Answer: n° (%)	Answer: n° (%)
Importance of cardiac imaging training	Unimportant: 1 (0%)	Slightly important: 14 (6%)	Moderately important: 75 (32%)	Important: 90 (38%)	Very important: 55 (24%)
Adequateness of CCTA imaging training	Definitely Not: 26 (11%)	Probably Not: 40 (17%)	Possibly: 59 (25%)	Probably: 50 (21%)	Definitely: 60 (26%)
Adequateness of CMR imaging training	Definitely Not: 28 (12%)	Probably Not: 42 (18%)	Possibly: 69 (29%)	Probably: 46 (20%)	Definitely: 50 (21%)
Best teaching method (regarding the reporting)	Alone with correction: 80 (34%)	Assisted reading: 135 (66%)	Just watching report: 0 (0%)		
Preference about training	Didactic lecture: 47 (20%)	Conference: 4 (2%)	Didactic lecture + Conference: 184 (78%)		
Possibility of research in cardiac imaging	Never: 16 (7%)	Rarely: 39 (17%)	Sometimes: 63 (27%)	Often: 67 (29%)	Always: 50 (20%)
Importance of doing research in cardiac imaging	Unimportant: 7 (3%)	Slightly Important: 17 (7%)	Moderately Important: 64 (27%)	Important: 80 (34%)	Very Important: 67 (29%)
Work in cardiac imaging in the future	Definitely Not: 30 (13%)	Probably Not: 31 (13%)	Possibly: 81 (35%)	Probably: 49 (21%)	Definitely: 44 (18%)
Relationships between cardiology and radiology	Good: 111 (47%)	Not good: 32 (14%)	Unsure: 92 (39%)		
In the future cardiac imaging will be done by	Radiologist: 39 (17%)	Combined Readouts: 147 (63%)	Radiologist extracardiac findings only: 6 (3%)	Cardiologist: 24 (10%)	Unsure: 19 (7%)

CCTA: coronary computed tomography angiography; CMR: cardiac magnetic resonance

## Discussion

This survey aimed at investigating the educational offerings in cardiac imaging in Italian University hospitals, as well as exploring the vision of the residents regarding both the present and the future of this subspecialty. Collected data provided an updated snapshot of cardiac imaging practice and education in Italy, which is likely applicable also to similar European countries.

### Technological profile in Italian academic centers and training possibilities

National measures to contain health care costs in Italy brought to a progressive obsolescence of CT/MR equipment in recent years, enhanced by the fact that the country is the second in Europe in terms of CT and MR scanners installed [14, 15]. Nevertheless, the results of our survey showed that the minimum standard requirements to perform CCTA and CMR were fulfilled (i.e., 64-slice scanner CT and ≥ 1.5 T magnets equipped with chest multielement radiofrequency coils and an ECG monitoring system) in all Italian academic centers with residency programs accreditation. This is the natural evolution of data from two previous surveys of the Italian Cardiac Radiology Society, which reported that more than 96% of CCTAs were performed with at least a 64-slices scanner CT [16] and nearly 100% of CMRs were performed with at least a 1.5 T scanner [17].

However, cardiac imaging education is limited to basic applications in about the half of the sites. On this regard, the paradigm of CMR is particularly significant. In 45% of the centers involved in the study, advanced CMR imaging techniques are not routinely performed: 4D-flow is used in only 12% of the centers, but also a state-of-the-art technique, which should enter in daily routine, like myocardial mapping, is performed in only 43% of the centers. This can likely reflect the average obsolescence of MR scanners in the national territory (i.e., more than 5 years) which reaches up to 51% [18].

### Number of examinations performed and skills

The Cardiovascular Radiology Residency Training Program in Italy spans a period of 6 months to be covered within 4 academic years. The goal would be, for each resident, to achieve independent competency and to continue self-education, as well as life-long learning techniques.

On this regard, we found significant differences in training opportunities in Italy between CCTA and CMR. The amount of CCTA scans performed per week was highly variable, ranging from 1 to 10 (in 50% of the centers) to more than 20 (in approximately 25% of the centers). CMR practice resulted in an average number of 5 performed exams per week, corresponding to an average of approximately 100–150 observed cases per resident during the training period. Considering the average number of weekly examinations performed in the evaluated centers for both CCTA and CMR, it is possible to learn about the total amount of examinations developed throughout the entire period of the residency course, which proves useful to sit to the EBCR Diploma examination [5].

In the majority of centers, radiologists administered drugs independently, which confirms the evidence that a radiologist could handle cardiac imaging procedures from acquisition to reporting.

**Fig. 2:**
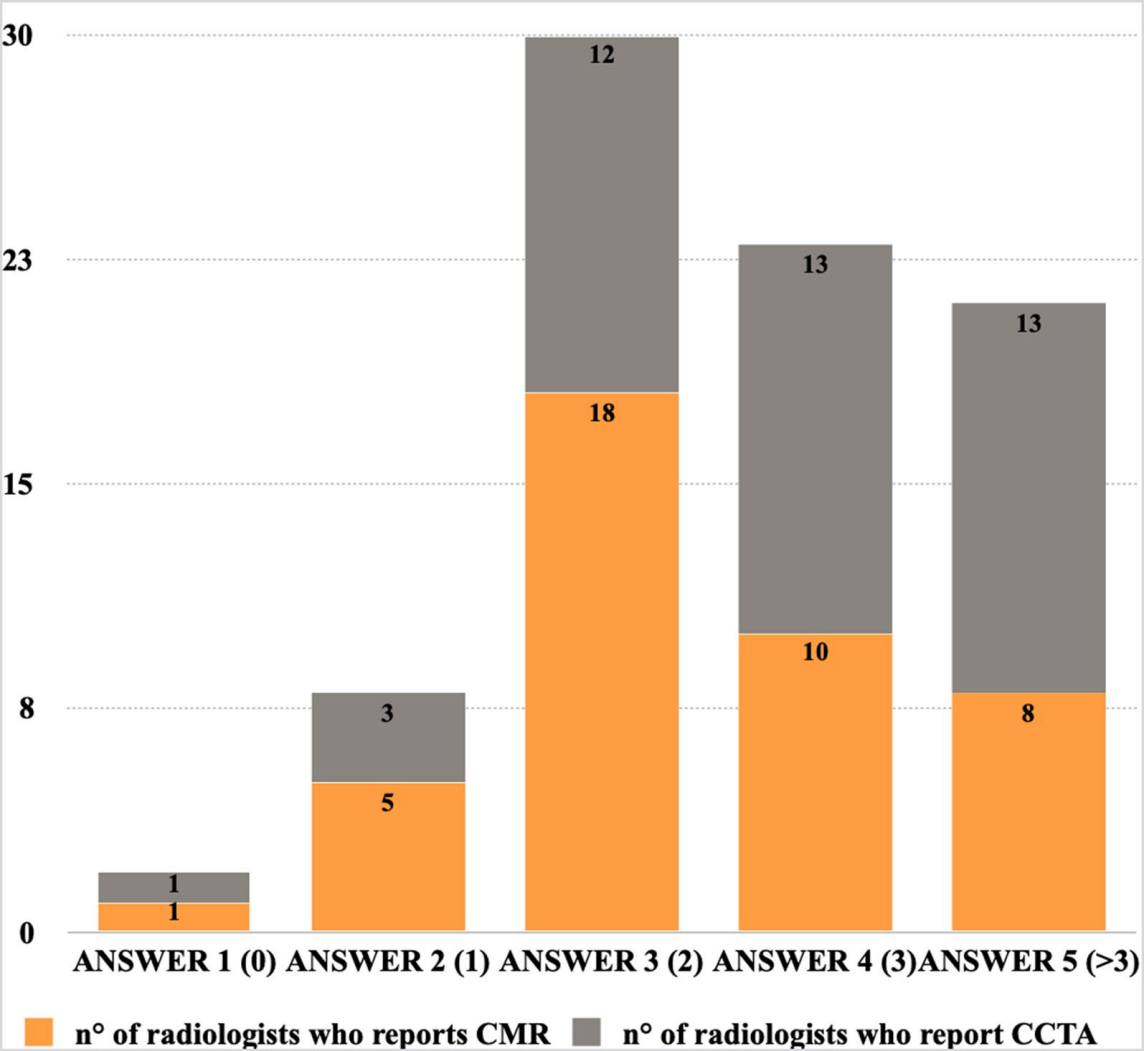
n° of radiologists who report CMR and CCTA. Answer 1 (0 radiologists), answer 2 (1 radiologist), answer 3 (2 radiologists), answer 4 (3 radiologists), answer 5 (> 3 radiologists)

Furthermore, the data make it easy to affirm that most radiologists involved in cardiac imaging perform CCTA, if compared to CMR (Fig. 2); CMR education represents a more challenging field and it will, therefore, be essential to improve primarily the skills of CMR during the future trainings.

### Educational program aspects and future perspectives

Italian educational system is not structured in level 1–3 courses, as residency program is the “entry level” to start cardiac imaging practice, without a formal accreditation exam. This is eventually followed by a research postdoc or PhD.

Subspecialty competence in cardiac imaging must be provided to radiology residents as stated by the Ministry of Education, University and Research’s guidelines. This is in contradiction with our observed data, showing that 25% of Residency Programs lack the presence of a complete theoretical and practical educational training. However, probably at least a part of the residents of these schools have the opportunity to complete their training attending different schools and hospitals during the residency program, using the way of the external training, included in the Residency Training Program in Italy.

Most participants (74%) considered cardiac radiology relevant in their future careers but, at the same time, 28–30% of them reported the perception of an inadequate training.

Looking at our survey’s data, one may speculate that inefficient training is mostly center based (i.e., attributable to the suboptimal educational offer) rather than an organizational failure of the training programs.

Where cardiac imaging rotation is nonexistent, radiologists should be offered the opportunity of completing their cardiac rotation in referral cardiac centers.

Another important educational opportunity offered to the Italian radiology residents is the wide offer of the Italian College of Cardiac Radiology by SIRM in terms of basic and advanced theoretical and practical courses (e.g., “ABCardio” and “focus on” courses). This Italian educational offer is further integrated by the international initiatives of the ESCR (ESCR educational webinars: “basic,” “advance” and “case based”) and the European School of Radiology (ESOR) (i.e., ESOR Foundation Courses and ESOR Galen Courses).

Furthermore, in response to the COVID-19 pandemic, webinar educational activity was further expanded with monthly meetings (called “For beginners, Technical updates, Clinical-radiological correlation and Live Cases”). These always-available online resources seem to be an excellent tool for integrating and enhancing educational activities, and their availability and implementation will have to continue even when face-to-face meetings can be resumed.

Overall, our results are comparable to a similar survey focusing on cardiac imaging training from the USA [13]. In the US survey, 71% of the residents had at least one dedicated cardiac imaging rotation of 3.37 weeks, building an overall experience in cardiac imaging of approximately 50–60 examinations during the entire period.

It is clear that the current programs not always combine cardiac examinations together with lectures, and alternative pathways should be sought by academic centers in order to provide a more complete cardiac educational route. Furthermore, a recent survey [19], looking at the barriers to academic activities that cardiovascular radiology trainees face worldwide, underlined the need for an update of the training programs and underlined the relevant role of academic activities in the cardiovascular department.

63% of radiologist residents imagine believe in a strong collaboration between radiologists and cardiologists in the field of cardiac imaging. These results are of utmost importance and highlights the fact that the future generations recognize the importance of a multidisciplinary approach to medicine, have a willingness to cooperate and an already well-established propensity to work in multidisciplinary clinical teams.

The UEMS recently approved the new ‘European training requirements in cardiology’ [20], where cardiologists are required to achieve a level of independence of 3 (i.e., trainee is able to perform the activity under indirect supervision) in cardiac imaging. This boosts an urgent adjustment of training process in cardiac imaging, in order not to transform the radiologist into a supporting figure to the imaging workflow.

However, it is nonetheless important to underline that in Italy, according to our data, only in 10% of CCTA and in 18% of CMR, there is a radiology-cardiology combined readout and these rather low percentages recognize some answers and open-up possible scenarios:

- In Italy, radiologist is the only professional figure allowed to perform and report CCTA or CMR [21, 22];
- Impact on cost-effectiveness of two professional figures on a single imaging modality remains questionable, also in the light of the advance cardiac imaging reimbursement issues all over the world;
- Regardless of a radiology-cardiology combined readout, a cardiologist at the occurrence can be involved according to a local expertise and to an organization collaborating with the radiologist in a patient-centered approach;

Similar data are confirmed by the ESCR, the MR/CT registry [23] which is the largest of ongoing data collections with over 340.000 cases included at the time of writing the manuscript, where a consensus reading of 18% for CCTA and 27% for CMR is reported.

Considering the “cardiac-imaging tsunami” that is approaching, the presence of a “clinical” radiologist, well trained in the field of cardiac imaging, will be fundamental. In patients with coronary artery disease, the radiologist can evaluate the heart and thorax beyond the coronary arteries and suggest an ischemia test (Stress CMR or CT perfusion) or, in patients with structural heart disease, he might evaluate the aortic, mitral valves, as well as the vascular accesses. In CMR, he would be able to optimize the acquisition protocol and provide imaging findings that are useful for the clinical management of patients.

This research has some limitations though. Firstly, while all Italian Universities responded to the first part of the survey, we only obtained replies from a moderate percentage of the Italian residents regarding the second part. This was probably due to the fact that the latter was not addressed directly to each resident but shared via social media; hence, the answers may not represent the global sample, being potentially biased by the selection of physicians who are likely to be interested in cardiac imaging. Secondly, the survey was focused on radiology activity, only offering a partial perspective of cardiac imaging training in Italy, with an obvious underestimation on the number and percentage of examinations performed by the cardiologists. Thirdly, the manuscript mainly focused on the training of radiologists, with no mention of the training of cardiologists; further studies could be needed to compare the training in advanced imaging of both categories. Finally, the survey does not assess several training opportunities offered to radiology residents outside from the specific site of each school. Hence, this survey may partially underestimate the true educational offer in cardiac imaging provided to the radiology residents.

## Conclusions

All centers met the technical requirements for cardiac imaging, limiting its use to basic applications in roughly half of the cases, and thus our study highlighted the need for technological renewal to support the introduction of the most advanced state-of-the-art cardiac imaging techniques in academic centers. Furthermore, we highlighted a significant awareness of radiology residents regarding the importance of cardiac imaging in their training curriculum: The implementation of an educational network might be the key for supporting the growth of this subspecialty field. Finally, we emphasized the importance of multidisciplinary medical teams as a tool for improving patient care quality and value.

## Supplementary Information

**Additional file 1**. First part of the survey (questions on CCTA and MR diagnostic services); second part of the survey (questions to all radiologist residents).

## Data Availability

Availability by mailing the corresponding authors.

## Abbreviations

CCTA: Coronary computed tomography angiography
CMR: Cardiac magnetic resonance
CT: Computed tomography
EBCR: European Board of Cardiovascular Radiology
ESCR YC: European Society of Cardiovascular Radiology Young Club
ESR: European Society of Radiology
FFR-CT: Fractional floe reserve-computed tomography
MR: Magnetic resonance
SCCT: Society of Cardiovascular Computed Tomography
SIRM: Italian Society of Medical and Interventional Radiology
TAVI: Transcatheter aortic valve implantation

## References

[1] Charles E, Zorana M, Geoffrey C (2018). Recent trends in utilization of cardiovascular imaging and invasive coronary angiography in the large scale united states military healthcare system. J Am Coll Cardiol.

[2] Levin DC, Rao VM, Parker L (2005). Recent trends in utilization of cardiovascular imaging: how important are they for radiology?. J Am Coll Radiol.

[3] ESR European Training Curriculum Undergraduate Level (Edition 2017—Design 2018).pdf. In: European Society of Radiology. https://www.myesr.org/media/2843. Accessed 21 Nov 2020

[4] ESR European Training Curriculum Level III (2020).pdf. In: European Society of Radiology. https://www.myesr.org/media/2840. Accessed 21 Nov 2020

[5] EBCR (European Board of Cardiovascular Radiolog) Diploma. https://www.escr.org/diploma/ Accessed 25 Feb 2021

[6] EACCME®. https://eaccme.uems.eu/home.aspx. Accessed 25 Feb 2021

[7] Esposito A, Gallone G, Palmisano A (2020). The current landscape of imaging recommendations in cardiovascular clinical guidelines: toward an imaging-guided precision medicine. Radiol Med.

[8] Pontone G, Di Cesare E, Castelletti S (2021). Appropriate use criteria for cardiovascular magnetic resonance imaging (CMR): SIC-SIRM position paper part 1 (ischemic and congenital heart diseases, cardio-oncology, cardiac masses and heart transplant). Radiol Med.

[9] Francone M, Aquaro GD, Barison A (2021). Appropriate use criteria for cardiovascular MRI: SIC—SIRM position paper Part 2 (myocarditis, pericardial disease, cardiomyopathies and valvular heart disease). J Cardiovasc Med (Hagerstown).

[10] Esposito A, Francone M, Andreini D (2021). SIRM-SIC appropriateness criteria for the use of Cardiac Computed Tomography. Part 1: Congenital heart diseases, primary prevention, risk assessment before surgery, suspected CAD in symptomatic patients, plaque and epicardial adipose tissue characterization, and functional assessment of stenosis. Radiol Med.

[11] European Society of Cardiovascular Radiology Young Club. https://www.escr.org/society/#young-club. Accessed 25 Feb 2021

[12] Green GE, Forman HP (2006). Residency training as technology matures a survey of radiology residents’ training experiences. Acad Radiol.

[13] Minocha J, Yaghmai V, Hammond N (2010). Cardiac imaging training in radiology residency programs: a survey of radiology chief residents. Acad Radiol.

[14] Fernández Lozano I, Pozo Osinalde E, García Bolao I (2018). Criteria for the management of technological assets in cardiovascular imaging. Rev Esp Cardiol (Engl Ed).

[15] COCIR Medical Imaging Equipment Age Profile & Density—2019 Edition. https://www.cocir.org/media-centre/publications/article/cocir-medical-imaging-equipment-age-profile-density-2019-edition.html. Accessed 21 Nov 2020

[16] Cademartiri F, Di Cesare E, Francone M (2015). Italian registry of cardiac computed tomography. Radiol Med.

[17] Francone M, Di Cesare E, Cademartiri F (2014). Italian registry of cardiac magnetic resonance. Eur J Radiol.

[18] Parco Tecnologie Diagnostiche Per Immagini. https://www.confindustriadm.it/wp-content/uploads/2021/03/Infografica-OPI.pdf. Accessed 20 Jul 2021

[19] Arzanauskaite M, Shelmerdine S, Choa JMD (2021). Academia in cardiovascular radiology: are we doing enough for the future of the subspecialty?. Clin Radiol.

[20] European training requirements in cardiology. https://www.uems.eu/media-and-library/documents/adopted-documents/2020. Accessed 25 Feb 2021

[21] Gazzetta Ufficiale. https://www.gazzettaufficiale.it/eli/id/2020/08/12/20G00121/sg. Accessed 25 Feb 2021

[22] Gazzetta Ufficiale. https://www.gazzettaufficiale.it/eli/id/2018/10/10/18A06555/sg. Accessed 25 Feb 2021

[23] MR/CT Registry - STARTPAGE. https://www.mrct-registry.org/. Accessed 21 Jan 2020

