# Supplementary material for: Challenges and opportunities to delivering cardiac imaging training: a national survey by the Italian college of cardiac radiology
Source: Insights Imaging. 2021 Sep 27;12:136. doi: 10.1186/s13244-021-01076-5 (PMC8475361; doi:10.1186/s13244-021-01076-5)
Supplement: Supplementary file 1 — Additional file 1. First part of the survey (questions on CCTA and MR diagnostic services); second part of the survey (questions to all radiologist residents). [file 13244_2021_1076_MOESM1_ESM.docx]

**ELECTRONIC SUPPLEMENTARY MATERIAL**

*Questions on CCTA and MR diagnostic services*

1. How many slices does the best performing scanner used for CCTA exams have in your university medical facilities center?
2. 64 slices
3. 128 slices
4. 256/320 slices – whole heart coverage
5. How many CCTA scans (excluding pre-TAVI scan) are performed each week?
6. 0
7. 1-10
8. 11-20
9. >20
10. How many pre-TAVI scans are performed each week?
11. 0
12. 1-5
13. 5-10
14. >10
15. CCTA scan are accompanied by an “advanced” cardio-radiological imaging evaluation:
16. None
17. Dual-Energy
18. Delayed enhancement
19. Stress perfusion
20. FFR-CT
21. CCTA scans in which type of patient is it required?
22. Outpatients
23. Inpatients
24. Outpatients + inpatients
25. Outpatients + inpatients + emergency room
26. N° of radiologist performing CCTA at the specialization school that you attend in your University medical facilities center:
27. 0
28. 1
29. 2
30. 3
31. >3
32. Percentage of radiologist performing CCTA:
33. 0-24%
34. 25-49%
35. 50-74%
36. 75-100%
37. CCTA experience of the senior radiologist:
38. <5 years
39. >5 years
40. Are beta-blockers and nitrates administered during the CCTA scan?
41. Yes, independently by the radiologist
42. Yes, in collaboration with the cardiologist
43. No
44. Who reports CCTA?
45. Radiologist
46. Cardiologist
47. Radiologist + Cardiologist
48. Are there radiology technicians dedicated to CCTA?
49. Yes
50. No
51. What field intensity has the best performing scanner in your University medical facilities center used for CMR scans?
52. <1.5T
53. 1.5T
54. 3T
55. How many channels has the coil that you use for CMR scans?
56. <8 channels
57. 8 channels
58. 16 channels
59. 32 channels
60. <32 channels
61. How many CMR exams (excluding MR-angiography) are performed each week?
62. 0
63. 1-5
64. 5-10
65. >10
66. How many MR-angiography exams are performed each week?
67. 0
68. 1-5
69. 5-10
70. >10
71. In which type of patients are CMR exams required?
72. Outpatients
73. Inpatients
74. Outpatients + inpatients
75. CMR scan are accompanied by an “advanced” cardio-radiological imaging evaluation:
76. None
77. Mapping sequences
78. Stress perfusion
79. 4D-Flow
80. N° of radiologist performing CMR:
81. 0
82. 1
83. 2
84. 3
85. >3
86. Percentage of radiologist performing CMR:
87. 0-24%
88. 25-49%
89. 50-74%
90. 75-100%
91. Senior radiologist experience:
92. <5 years
93. >5 years
94. Is cardiology assistance normally present during CMR exams:
95. Yes
96. No
97. Who reports CMR?
98. Radiologist
99. Cardiologist
100. Radiologist + cardiologist
101. Are there radiology technicians dedicated to CMR?
102. Yes
103. No
104. Are cardio-radiology didactic lectures included in the specialist training course?
105. No
106. CCTA
107. CMR
108. CCTA – CMR
109. How many hours of didactic lectures per year dedicated to cardiac imaging are done during the specialty school?
110. <5 hours
111. 6-10 hours
112. 10-15 hours
113. >15 hours
114. How many hours of congress per year dedicated to cardiac imaging can be followed by radiology residents in your school?
115. <5 hours
116. 6-10 hours
117. 10-15 hours
118. >15 hours
119. Is there the possibility to do a training course/rotation dedicated to cardiac imaging?
120. It exists, and is inserted by “default” for all radiology residents
121. It exists, but is not inserted by “default” for all radiology residents
122. It does not exist, but we are given the opportunity to do so in other centers
123. It does not exist
124. The dedicated specialization path includes:
125. Dedicated specialization path does not exist
126. CCTA
127. CMR
128. CCTA and CMR
129. Involvement in the reporting of cardio-radiological exams:
130. Just watching reports
131. Assisted reading
132. Alone reading with correction
133. Are cardiac imaging cases logbooks kept in the specialist training course in order to allow an independent individual study?
134. Yes
135. No
136. Percentage of radiology resident dedicated to cardiac imaging:
137. <25%
138. 25-50%
139. 50-70%
140. 70-100%

*Questions to all radiologist residents*

1. In your opinion, nowadays, how important is it to have a good cardio-radiological imaging training from 1 (irrelevant) to 5 (fundamental)?
2. 1
3. 2
4. 3
5. 4
6. 5
7. Do you believe that you receive / have the possibility to receive adequate training in CCTA from 1 (absolutely not) to 5 (absolutely yes)?
8. 1
9. 2
10. 3
11. 4
12. 5
13. Do you believe that you receive / have the possibility to receive adequate training in CMR from 1 (absolutely not) to 5 (absolutely yes)?
14. 1
15. 2
16. 3
17. 4
18. 5
19. Which do you believe is the best method of “teaching” the reporting of cardioradiological exams?
20. Just watching reports
21. Assisted reading
22. Alone reading with correction
23. What you prefer as cardioradiological training?
    1. Didactic lecture
    2. Conference
    3. 1+2
24. Is there the possibility in your medical facilities centre to do research in cardioradiological imaging from 1 (absolutely not) to 5 (very much)?
25. 1
26. 2
27. 3
28. 4
29. 5
30. Do you believe it is / would be important to have the possibility to do research in cardioradiological imaging from 1 (absolutely not) to 5 (very much)?
31. 1
32. 2
33. 3
34. 4
35. 5
36. Do you plan to also do cardiac imaging 1 (absolutely not) to 5 (absolutely yes) in your future?
37. 1
38. 2
39. 3
40. 4
41. 5
42. What is the current professional working relationship between radiologist - cardiologist in your University?
43. Good
44. Not good
45. Unsure
46. From your point of view, what do you believe to be the future of cardiac imaging?
47. It will be solely in the hands of radiologists
48. It will be a combined radiologist-cardiologist reading and analysis
49. The radiologist will only deal with the extracardiac findings
50. Unsure
